# Identification of Salivary Gland Proteins Depleted after Blood Feeding in the Malaria Vector *Anopheles campestris*-like Mosquitoes (Diptera: Culicidae)

**DOI:** 10.1371/journal.pone.0090809

**Published:** 2014-03-05

**Authors:** Sriwatapron Sor-suwan, Narissara Jariyapan, Sittiruk Roytrakul, Atchara Paemanee, Atchara Phumee, Benjarat Phattanawiboon, Nuchpicha Intakhan, Wetpisit Chanmol, Paul A. Bates, Atiporn Saeung, Wej Choochote

**Affiliations:** 1 Department of Parasitology, Faculty of Medicine, Chiang Mai University, Chiang Mai, Thailand; 2 National Center for Genetic Engineering and Biotechnology (BIOTEC), National Science and Technology Development Agency, Pathumthani, Thailand; 3 Department of Parasitology, Faculty of Medicine, Chulalongkorn University, Bangkok, Thailand; 4 Division of Biomedical and Life Sciences, Faculty of Health and Medicine, Lancaster University, Lancaster, United Kingdom; Metabiota, United States of America

## Abstract

Malaria sporozoites must invade the salivary glands of mosquitoes for maturation before transmission to vertebrate hosts. The duration of the sporogonic cycle within the mosquitoes ranges from 10 to 21 days depending on the parasite species and temperature. During blood feeding salivary gland proteins are injected into the vertebrate host, along with malaria sporozoites in the case of an infected mosquito. To identify salivary gland proteins depleted after blood feeding of female *Anopheles campestris*-like, a potential malaria vector of *Plasmodium vivax* in Thailand, two-dimensional gel electrophoresis and nano-liquid chromatography-mass spectrometry techniques were used. Results showed that 19 major proteins were significantly depleted in three to four day-old mosquitoes fed on a first blood meal. For the mosquitoes fed the second blood meal on day 14 after the first blood meal, 14 major proteins were significantly decreased in amount. The significantly depleted proteins in both groups included apyrase, 5′-nucleotidase/apyrase, D7, D7-related 1, short form D7r1, gSG6, anti-platelet protein, serine/threonine-protein kinase rio3, putative sil1, cyclophilin A, hypothetical protein Phum_PHUM512530, AGAP007618-PA, and two non-significant hit proteins. To our knowledge, this study presents for the first time the salivary gland proteins that are involved in the second blood feeding on the day corresponding to the transmission period of the sporozoites to new mammalian hosts. This information serves as a basis for future work concerning the possible role of these proteins in the parasite transmission and the physiological processes that occur during the blood feeding.

## Introduction

Malaria remains one of the most important infectious diseases in the world, and despite some progress in control has re-emerged in tropical regions that have experienced rapid population growth [1], [2]. Transmission of malaria parasites, various *Plasmodium* species, is typically via the bites of *Anopheles* mosquito vectors. A mosquito must blood feed at least twice to transmit malaria, once to acquire the malaria parasites and once to deliver the infective sporozoite stages to a new host. Female mosquitoes become infected with *Plasmodium* through taking a blood meal from an infected person, taking up gametocyte forms along with the blood meal. The parasites leave the lumen of the midgut and then develop as oocysts in the midgut wall for a week or more, before the resulting sporozoite stages are released into the haemocoel and travel to the salivary glands of the mosquito [3]. Malaria sporozoites must invade the salivary glands for maturation before transmission to vertebrate hosts in the saliva. When the mosquito next takes a blood meal, these parasites are mixed with the saliva and injected with the bite, and the transmission of malaria is complete. Therefore, infection with malaria is always accompanied by injection of salivary proteins. The time required for development in the mosquito vector (the duration of the sporogonic cycle) ranges from 10 to 21 days, depending on the parasite species and the environmental temperature. During this period mosquitoes will usually take additional blood meals, perhaps every 2–3 days, before it becomes capable of transmitting malaria. If a female mosquito vector does not survive longer than the duration of the sporogonic cycle, then it will not be able to transmit malaria parasites [3].

Mosquito saliva contains a cocktail of substances to help blood feeding, including anticoagulants, anti-inflammatory and immunosuppressive factors [4], [5]. During blood feeding, depletion of salivary proteins from the female glands occurs continuously as mosquitoes feed to repletion. A reduction in total salivary gland proteins from the vectors *Anopheles stephensi*, *An. albimanus*, *An. gambiae* and *An. freeborni* after blood-feeding on human volunteers and hamsters has been observed previously [6]. In *An. gambiae*, the gSG6 protein is secreted with the saliva while the female mosquito probes for feeding [7], whilst others have reported that prior to and after blood feeding, 52 and 41 salivary gland transcripts of female *An. gambiae* are significantly up-regulated and down-regulated, respectively [8]. A proteomic study of salivary glands of *Aedes aegypti* has revealed several salivary gland proteins up-regulated ten days post feeding, for example, apyrase, D7 protein, salivary serpin putative anticoagulant, and putative 30 kDa allergen-like protein [9]. Changes in the amount of total salivary gland proteins and/or electrophoretic protein profiles after the first blood meal have also been reported for several other mosquito species, for example, *Aedes caspius*, *Armigeres subalbatus*, *Culex pipiens* and *Mansonia uniformis* [10]–[12].

Recently, salivary gland proteins of female *An. campestris*-like mosquitoes, a potential malaria vector of *Plasmodium vivax* in Thailand [13], [14], were analyzed for the first time [15]. This study showed the presence of five major salivary gland proteins presumed to be involved in blood feeding, a putative 5′-nucleotidase/apyrase, anti-platelet protein, long form D7 salivary protein, D7-related 1 protein, and gSG6. As described above, various other studies have analysed changes in mosquito salivary gland composition following a single blood meal. In contrast, there is little information on changes in composition after a second or subsequent blood meal in *Anopheles* mosquitoes. However, given that a female mosquito will feed two or more times before transmitting malaria sporozoites, such analyses are important for understanding the transmission of malaria. Therefore, in the current study depletion of *An. campestris*-like salivary proteins after both the first and second blood meals was analysed and the composition compared using two-dimensional gel electrophoresis (2-DE) coupled with nano-liquid chromatography-mass spectrometry (NanoLC-MS). The results revealed significant compositional differences in salivary gland proteins present after one blood meal compared with after two blood meals, this taking place 14 days later when the sporogonic cycle would have completed in an infected mosquito.

## Results

### Comparison of Female Salivary Gland Protein Profiles between Unfed and Blood Fed *Anopheles campestris*-like Mosquitoes

**Figure 1:**
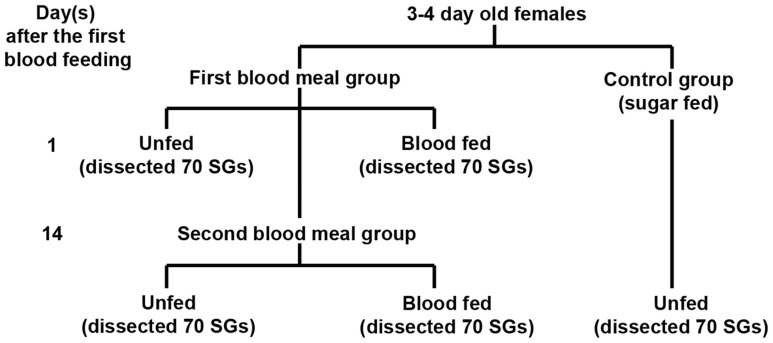
Experimental design for the first and the second blood feeding of *An. campestris-*like.

**Figure 2:**
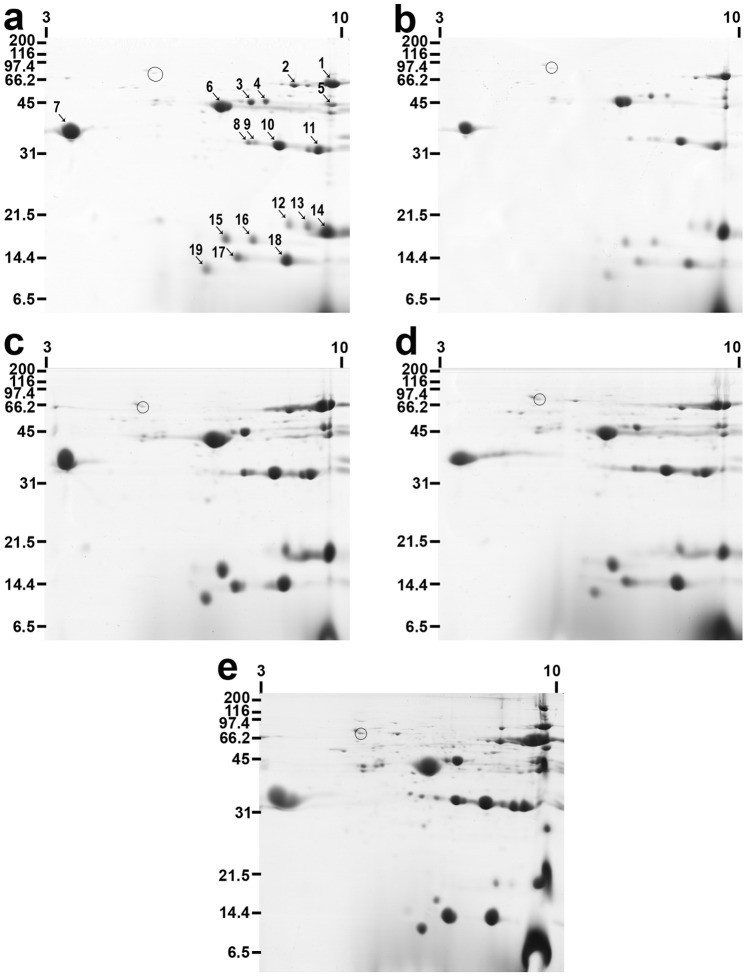
Comparison of 2-DE protein profiles of female salivary gland proteins of *An. campestris-*like. Proteins were separated on Immobiline Dry Strips 7–10. Separation in the second dimension was performed using 15% SDS-PAGE. The gels were stained with Coomassie blue. Molecular mass markers are indicated on the left in kDa. Isoelectric points (pI) are indicated at the top. Numbers indicate major salivary gland proteins. **a** representative of 2-DE gels of salivary gland proteins of unfed females from the first blood meal group; **b** salivary gland proteins of blood-fed females the first blood meal group; **c** salivary gland proteins of unfed females from the second blood meal group; **d** salivary gland proteins of blood-fed females the second blood meal group; **e** salivary gland proteins of unfed females from sugar fed control group. Circle indicates an internal control protein.

The experimental design followed is illustrated in Figure 1, each experiment yielding five groups of mosquitoes, and each group containing 70 individuals: sugar-fed, taken after one blood meal, control for one blood meal, taken after two blood meals, and control for two blood meals. This experiment was performed three times, each experiment produced essentially identical results, and representative results from one experiment are shown in Figure 2. Analysis by 2-DE showed that female *An. campestris*–like salivary glands contained 19 major proteins (Fig. 2a). The molecular mass of these well-resolved spots varied from 10–72 kDa, with pI ranging from 3.9–10. To identify them, each major spot was excised, digested with trypsin, and subjected to NanoLC-MS analysis (Table 1, Table S1). Fourteen of the 19 spots could be assigned an identity, these including apyrase, which was spot number 1 (SN1), 5′nucleotidase/apyrase (SN2), anti-platelet protein (SN7), D7 (SN10), D7-related 1 (SN12), and gSG6 (SN19). Comparing the protein profiles of female salivary glands of the unfed controls (Fig. 2a) and the blood fed mosquitoes from the first blood meal group (Fig. 2b) they were essentially identical regarding the number of proteins detected. However, there were significant differences in the density of the protein spots, in the blood fed mosquitoes the density of all the major protein spots was reduced compared to the unfed controls. A heat shock cognate 70 kDa protein of *Ae. aegypti* (accession number gi|94468966) was used as an internal control in 2-DE gels (Table 1, 2, Fig. 2). This protein is circled in Figure 2a – e and showed no significant difference in density between samples. To provide a quantitative measure of depletion in the major proteins, gel imaging analysis was performed (Table 2). The amount of protein depletion varied from spot to spot (0.13–7.15) and whilst these values are not directly comparable due to potential differences in the staining of individual proteins, they do suggest differential delivery of salivary gland proteins during blood feeding. This was supported by analysis of the % of each protein depleted, revealing a range of values from SN2, which showed the highest depletion at 79.71%, to SN14, which showed 13.18% depletion. However, excepting SN6, 14, and 19, the remaining 16 proteins showed depletion of approximately 50% or greater. Thus the taking of one blood meal significantly depleted the salivary gland contents, but individual proteins were depleted to differing extents.

**Table 1:** A list of major protein spots significantly decreased in volume immediately after the first blood feeding in female *An. campestris*-like salivary glands identified by NanoLC-MS.

SN^a^	Accessionnumber^b^	Protein description[species]	Proteinscore^d^	No. of peptides/% coverage	DatabaseMw/pI	ObservedMw/pI	Peptide Sequences
1	gi|4582524	apyrase [*An. gambiae*]	31	1/1	62.1/8.9	68/9.8	R.VFHTVQELR.K
2	gi|208657633	putative 5′-nucleotidase/apyrase[*An. darlingi*]	68	2/6	63.5/8.7	67/8.0	K.NVPDQSFPLTLIHINDLHAR.FK.QLPPDAMTLGNHEFDHSPK.G
3	gi|347968035	AGAP002538-PA[*An. gambiae* str. PEST]	37	1/2	36.5/5.5	56/7.5	R.NLTTDELSR.L
4	gi|170033361	conserved hypothetical protein[*Cx. quinquefasciatus*]	41	1/2	38.6/5.6	55/7.2	R.NDDLHDIER.F
5	gi|242021351	hypothetical proteinPhum_PHUM512530 [*Pediculus humanus corporis*]	35	1/3	33.7/9.4	55/9.8	R.DLNLLTPTSM.-
6	gi|170046888	serine/threonine-protein kinase rio3[*Cx. quinquefasciatus*]	33	2/5	56.9/9.4	52/6.7	R.SRLSGAASCDHR.SR.VGYKVNEDGEMVTK.H
7	gi|190576759	anti-platelet protein [*An. gambiae*]	40	1/4	27.2/4.1	38/4.1	R.EQELSDCIVDKR.D
8	NSH^c^					37/7.1	
9	gi|241998444	sil1, putative [*Ixodes scapularis*]	35	1/2	36.4/5.1	37/7.3	R.LNLETGRR.E
10	gi|15718081	D7 protein [*An. stephensi*]	41	1/3	36.9/8.8	36/7.7	R.QLYHGTVEGAAK.I
11	gi|158285343	AGAP007618-PA [*An. gambiae*str. PEST]	37	1/2	30.9/8.4	35/9.4	R.LADMMR.Q
12	gi|4538887	D7-related 1 protein [*An. gambiae*]	55	1/6	19.1/9.2	20/7.9	K.LIKPLNAIEK.D
13	gi|241616200	cyclophilin A, putative [*I. scapularis*]	41	1/4	22.1/9.2	20/9.0	K.FEDENFILK.H
14	gi|16225961	short form D7r1 salivary protein[*An. arabiensis*]	52	1/6	19/9.2	19/9.8	K.LIKPLNAIEK.D
15	gi|270014872	hypothetical protein TcasGA2_TC010859 [*Tribolium* *castaneum*]	31	1/4	16.1/7.8	17/6.8	-.MGDMQR.R
16	NSH					17/7.3	
17	NSH					14/7.0	
18	gi|312381960	hypothetical protein AND_05658[*An. darlingi*]	38	1/4	16.9/7.7	14/7.9	R.KSLEAMR.E
19	gi|13537666	gSG6 protein [*An. gambiae*]	87	2/10	13.7/5.3	12/6.5	R.DKVYCGHLDCTR.V K.VYCGHLDCTR.V
Control	gi|94468966	heat shock cognate 70[*Aedes aegypti*]	515	10/17	71.4/5.3	77.0/5.4	R.TTPSYVAFTDTER.L K.NQVAMNPTNTIFDAK.R K.DAGTISGLNVLR.IR.IINEPTAAAIAYGLDK.KR.IINEPTAAAIAYGLDKK.T R.FEELNADLFR.SK.ASIHDIVLVGGSTR.I K.LLQDFFNGK.EK.FELSGIPPAPR.G K.NALESYCFNMK.A

a Spot number refers to those shown in Fig. 2a.

b Accession number of the best hit of proteins from mosquitoes and/or arthropod species.

c NSH = not significant hit.

d Mowse score ≥30.

**Table 2:** Amounts of depletion of major salivary gland proteins from unfed mosquitoes and blood fed to repletion on mice.

SN^a^	First blood meal group					Second blood meal group			
	ASD ± SD^b^		Amountdepleted		%depletion	ASD ± SD		Amountdepleted	%depletion
	Unfed	Blood fed				Unfed	Blood fed		
1	4.16±0.08	1.78±0.07	2.38	57.11^c^		8.62±0.05	5.99±0.01	2.63	30.53^c^
2	0.55±0.03	0.11±0.01	0.44	79.71^c^		1.68±0.02	0.61±0.01	1.07	63.57^c^
3	0.70±0.01	0.30±0.01	0.4	56.87^c^		1.60±0.01	0.69±0.01	0.91	52.94^c^
4	0.46±0.01	0.12±0.01	0.34	74.38^c^		(−)^d^	(−)	(−)	(−)
5	0.59±0.02	0.24±0.01	0.35	59.16^c^		1.49±0.01	0.69±0.01	0.8	53.54^c^
6	4.61±0.06	3.42±0.02	1.19	25.96^c^		8.95±0.05	6.16±0.02	2.79	31.14^c^
7	10.31±0.08	3.16±0.05	7.15	69.35^c^		8.02±0.06	5.48±0.05	2.54	31.68^c^
8	0.26±0.01	0.05±0.01	0.21	78.89^c^		0.79±0.01	0.43±0.01	0.36	45.43^c^
9	0.22±0.01	0.09±0.01	0.13	57.06^c^		0.42±0.01	0.34±0.01	0.08	19.15^c^
10	2.67±0.08	1.38±0.01	1.29	48.19^c^		4.07±0.02	3.22±0.01	0.85	20.88^c^
11	4.40±0.04	1.43±0.03	2.97	67.41^c^		3.59±0.01	3.48±0.01	0.11	3.03
12	0.40±0.01	0.18±0.01	0.22	53.90^c^		4.95±0.03	2.74±0.02	2.21	45.10^c^
13	0.79±0.01	0.40±0.01	0.39	48.74^c^		1.77±0.01	0.76±0.01	1.01	56.86^c^
14	4.39±0.04	3.81±0.02	0.58	13.18^c^		4.90±0.06	2.69±0.02	2.21	45.10^c^
15	0.69±0.01	0.29±0.01	0.4	58.34^c^		2.20±0.02	1.93±0.02	0.27	12.34
16	0.53±0.01	0.23±0.01	0.3	56.53^c^		(−)	(−)	(−)	(−)
17	0.85±0.01	0.43±0.01	0.42	49.25^c^		1.85±0.01	1.37±0.01	0.48	26.27^c^
18	2.48±0.01	1.09±0.02	1.39	55.93^c^		4.81±0.02	4.34±0.02	0.47	9.72
19	0.64±0.01	0.37±0.01	0.27	41.78^c^		1.39±0.02	0.56±0.01	0.83	59.99^c^
Control	0.02±0.01	0.02±0.01	0	0		0.04±0.01	0.04±0.01	0	0

a Spot number refers to those shown in Fig. 2a.

b ASD ± SD = Average spot density ± Standard deviation.

c Student’s *t*-test, p≤0.05.

d Protein spot was absent.

### Comparison of Female Salivary Gland Protein Profiles after One Blood Feed but before Second Blood Feed

After taking one blood meal, the salivary gland protein content was replenished over the next 14 days, with 17 of the 19 major proteins increasing in amount again (Fig. 2c, Table 2). The two exceptions were SN4 (a conserved hypothetical protein) and SN16 (unidentified), which were not detected in mosquitoes prior to the second blood meal. Therefore these two components, which were depleted after one feed, did not recover but disappeared from the salivary glands, at least as major components. It should also be noted that they were absent from the sugar-fed control group (Fig. 2e), which was age matched for the second blood meal groups, indicating this may be age-related decline. After comparing the data from unfed three to four days old mosquito salivary glands with the glands of mosquitoes before a second blood feeding, all major protein spots were increased significantly. Similarly, when comparing this data with unfed 17–18 days old mosquito salivary glands, most of major protein spots were increased significantly, except SN 13, which was decreased significantly. Regarding the remaining majority of proteins that did increase significantly, in most cases these increases were such that the amounts present immediately before taking a second meal were actually much greater than before taking the first blood meal. For example, apyrase (SN1) had an average value of 4.16 before the first meal and 8.62 before the second meal (∼2 fold difference), and D7-related 1 (SN12) with a value of 0.4 before the first meal and 4.95 before the second meal (∼12 fold difference). Exceptions to these increases were SN7 (anti-platelet protein) and SN11, which were at slightly lower values, although still significantly replenished following the first blood meal.

### Comparison of Female Salivary Gland Protein Profiles before and after a Second Blood Feed in *Anopheles campestris*-like Mosquitoes

Comparison of the amount of each of the 17 major proteins present before and after the second blood meal again revealed statistically significant depletion of most protein spots, the exceptions being SN11, 15, and 18. Therefore, the remaining 14 salivary gland proteins are those predicted to accompany sporozoites from an infected mosquito. However, there were significant variations in the depletion of individual proteins comparing first and second blood meals. For example, SN11 showed a 67% depletion after the first blood meal, but only a 3% depletion after the second meal. For most proteins the % depletion after the second meal was lower than after the first meal, although for SN6, 13, 14, and 19 it was higher. However, because individual proteins were replenished to different extents following the first blood meal, these % values do not necessarily reflect the absolute amounts of protein delivered. For example, the amount of apyrase (SN1) depleted after the first meal (2.38) was similar to after the second meal (2.63). In contrast, the amount of anti-platelet protein (SN7) depleted was more after the first meal (7.15) compared to the second meal (2.54), whereas the amount of D7-related 1 (SN12) was less after the first meal (0.22) compared to the second (2.21). Overall these results indicate not only differential depletion and, therefore, delivery of individual salivary gland proteins within a blood feed, but also between first and second blood feeds.

## Discussion

Several studies have demonstrated changes in amount of total salivary gland proteins and/or one dimensional gel electrophoretic protein profiles after the first blood meal of various mosquito species, for example, *Ae. aegypti*, *Aedes caspius*, *An. stephensi*, *An. albimanus*, *An. gambiae*, *An. freeborni*, *Armigeres subalbatus*, *Cx. pipiens*, *Cx. quinquefasciatus*, and *Mn. uniformis* [6], [10]–[12]. However, only salivary gland proteins differentially expressed after the first blood feeding in *Ae. aegypti* have been identified [9]. Up-regulated salivary gland proteins ten days post blood feeding in *Ae. aegypti* include three D7 proteins, salivary apyrase, apyrase precursor, aldehyde dehydrogenase, salivary serpin putative anticoagulant, putative 30 kDa allergen-like protein, adenosine deaminase, 19.6 kDa secreted protein precursor, putative secreted protein, and a putative uncharacterized protein [9].

In addition, microarray transcriptome analyses of *Ae. aegypti* and *An. gambiae* salivary glands response to blood feeding have been performed [8], [16]. Down-regulated salivary gland transcripts of *Ae. aegypti* during the first three hours post-blood feeding have included an odorant binding protein, protease inhibitors, and immune genes [16]. Das et al (2010) [8] have reported that a small proportion of the salivary gland transcriptome of *An. gambiae* is dynamically changing already at two hours in response to blood feeding. The salivary gland transcripts encoding secretory proteins that displayed a lower abundance after blood feeding have included two OBPs (OBP 10 and OBP 7), two D7 long-form precursors (L1 and L2), two aminopeptidases, a trypsin 6 precursor, a salivary lipase, a 5′nucleotidase precursor, an apyrase, E1 protein, cecropin 3, defensin 1, and a hypothetical 6.2 precursor [8]. It is generally assumed that mRNA levels are a useful surrogate for protein concentration, however, mRNA levels do not necessarily correspond to concentration of proteins due to requirements for posttranslational modifications of some, and potential regulation of translation.

In this study, a proteomic approach, 2-DE coupled with NanoLC-MS, was used. Our study showed that 19 and 14 major salivary proteins of *An. campestris*-like females decreased in quantity after the first and the second blood meals, respectively. Significantly depleted proteins in the these groups included apyrase, 5′-nucleotidase/apyrase, D7, D7-related 1, short form D7r1, gSG6, anti-platelet protein, serine/threonine-protein kinase rio3, putative sil1, cyclophilin A, hypothetical protein Phum_PHUM512530, AGAP007618-PA, and two non-significant hit proteins, indicating that these polypeptides are introduced into the vertebrate hosts during blood feeding and facilitate the process of blood-feeding and potentially pathogen transmission. Our results, together with those from previous studies in *Ae. aegypti* and *An. gambiae* [8], [9], [16], indicate that proteins involved in hematophagy and pathogen transmission include salivary apyrase, 5′-nucleotidase/apyrase, D7 proteins, and putative 30 kDa allergen-like protein/anti-platelet protein.

Apyrase and 5′ nucleotidase proteins are known to facilitate the acquisition of a blood meal by the degradation of adenosine diphosphate (ADP), a mediator of platelet aggregation and inflammation [5] and prevent neutrophil activation [17]. Two genes of the 5′-nucleotidase family, putative 5′-nucleotidase and salivary apyrase, are expressed in the salivary glands of *An. gambiae* [18]. The sialotranscriptome of *An. darlingi* also presents evidence for the two orthologues, a full-length orthologue of the salivary 5′-nucleotidase of *An. gambiae* and a 5′-truncated clone best matching the *An. gambiae* salivary apyrase [19]. *An. gambiae* has been shown to require longer probing times during blood-feeding when an apyrase gene has been silenced [20]. It has also been reported that apyrase is reduced in *Plasmodium berghei*-infected *An. gambiae* mosquito salivary glands [21]. The reduction of apyrase levels in *P. gallinaceum* infected *Ae. aegypti* salivary glands caused an increase in mosquito probing time [22]. These studies indicate a role for apyrase and 5′ nucleotidase in increasing the time for *Plasmodium* transmission to a new vertebrate host. Our results showed that at least two proteins in the salivary glands of *An. campestris*-like mosquitoes matched apyrase and 5′-nucleotidase of other *Anopheles* mosquitoes. In addition, the most depleted protein spot of the first and second blood meal groups was SN2 matched with putative 5′-nucleotidase/apyrase [*An. darlingi*] suggesting that platelet aggregation might be the most vital mechanism used for blood feeding in *An. campestris*-like. Further studies on the role in facilitation of pathogen transmission of 5′-nucleotidase/apyrase in *An. campestris*-like should be performed.

Proteins of the D7 family are distantly related to the OBP (odorant binding proteins) super-family and present in the saliva or salivary glands of several female blood-sucking insects [23]–[25]. The D7 proteins are able to bind host biogenic amines such as serotonin and histamine to antagonize vasoconstrictor, platelet aggregating, and pain-inducing properties [26]. The D7 protein exists in two forms: a long form, which is found exclusively in mosquitoes and sand-flies, and the short forms, which are found in mosquitoes, sand-flies and other insects [25], [27]. Five D7-related short forms (D7r1, 2, 3, 4, and 5) and three D7 long forms have been identified in *An. gambiae* [18], [27]. The D7r1, 2, 3, 4 and D7 long forms have been shown to bind to the biogenic amines [18], [26]. Hamadarin, a short D7 protein 1 from *An. stephensi*, has been shown to inhibit the plasma contact system by preventing the activation of kallikrein by Factor XIIa [28]. By using a RNAi-mediated gene silencing method, it was shown that depletion of D7L2 resulted in decreased blood feeding capacity and as well as increased probing time [8]. Also the level of D7 related-1 protein precursor protein is decreased in *P. berghei*-infected salivary glands of *An. gambiae* [21]. The decreased production of D7 related-1 protein precursor may induce an increased local inflammatory response to mosquito bites, thus modifying the immune response to the parasite. In our study, a long form D7, matched best with a D7 protein [*An. stephensi*], and two D7 short forms, matched best with D7-related 1 protein [*An. gambiae*] and short form D7r1 salivary protein [*An. arabiensis*], were depleted significantly after blood-feeding. These results strongly support the involvement of D7 and D7 related-1 proteins in the blood feeding process.

gSG6, a small protein with unknown function, was first identified in *An. gambiae* females [29] and conserved in species members of the *An. gambiae* complex, i.e., *An. gambiae*, *An. melas*, *An. bwambae*, *An. quadriannulatus* A, and *An. arabiensis* [7]. These authors demonstrated that silencing of *An. gambiae* salivary gland gene, gSG6, results in increased probing time and reduced blood-feeding ability. Recently, *An. gambiae* gSG6, a reliable marker for exposure to *An. gambiae* bites [30]–[32], has been recently reported to be a good indicator for exposure to bites from three main African malaria vectors, i.e., *An. gambiae*, *An. arabiensis* and *An. funestus* [33]. In *An. campestris*-like, its gSG6 protein was depleted in both blood feeding groups with approximately 40–60% confirming that a least one of the gSG6 functions is involved in blood feeding. However, further studies are needed to characterize the biological properties of this unknown protein.

The 30 kDa/GE-rich/anti-platelet protein family was been first identified as a salivary antigen in *Ae. aegypti* and called 30-kDa allergen of *Ae. aegypti* [34]. The members of this family have been found in salivary transcriptomes and proteomes of both culicine and anopheline mosquitoes [35]–[38], where it has been named GE-rich protein. Later, a related unique anti-platelet protein, anopheline anti-platelet protein (AAPP), from the salivary gland of female *An. stephensi* was identified [39]. Several proteomic works have also indicated that the members of 30 kDa/GE-rich/anti-platelet protein family are one of the most abundantly expressed acidic proteins in the female salivary glands of mosquitoes studied so far [19], [35]–[38], [40], and also found in *An. campestris*-like [15]. Recently, members of the 30 kDa/GE-rich/anti-platelet family have been identified as antigens from four *Anopheles* species, i.e., *An. gambiae*, *An. albimanus*, *An. stephensi*, and *An. arabiensis*, and are thus potential candidates to serve as pan-*Anopheles* genus markers of immunological exposure [41]. A proteomic study of the salivary glands of *Ae. aegypti* has revealed that putative 30 kDa allergen-like salivary gland protein is up-regulated ten days post feeding [9]. In *An. campestris*-like, the anti-platelet protein was depleted in the blood feeding groups. The result suggested that one of the anti-platelet protein functions is involved in blood feeding. In addition, studying saliva allergens could provide valuable information on immune responses, with a view to developing new diagnostic tests for allergies to mosquito bites.

For serine/threonine-protein kinase rio3, putative sil1, cyclophilin A, hypothetical protein Phum_PHUM512530, AGAP007618-PA, and two non-significant hit proteins, no information on their function is available.

A chance of successful blood feeding of female mosquitoes depends on the efficiency of alteration of the host hemostatic response (platelet activation and aggregation, local vasoconstriction, and coagulation), inflammatory, and immune systems [5]. Results from the proteomic approach used in this study confirmed that *An. campestris*-like used at least anti-platelet aggregation (apyrase and anti-platelet protein), and anti-inflammatory (D7and D7-related) proteins to facilitate blood feeding. Although analysis in terms of putative functional association networks among the depleted proteins using STRING 9.0 Server [42], [43] (http://www.string-db.org) was performed in this study, no predicted confident association was reported (unpublished data). Further investigation on the functions of salivary gland proteins could be performed using transient RNAi gene-silencing assays on the salivary transcribed genes and examining how they influence the mosquito feeding and probing behavior on a vertebrate host and pathogen infected mosquitoes. Also the mechanism that would allow, for example, depletion of approximately 50% of one protein and 75% of another is unknown. However, it is known that individual proteins are not distributed evenly between different parts and lobes of salivary glands, so this may explain differential depletion. More studies on other mosquito species and using different methods of feeding, for example, artificial membrane feeding might provide some clues for explanation of the secretion mechanism in mosquito salivary glands.

Surprisingly, for *An. camprestris*-like, two protein spots (SN4 and 16) disappeared in the unfed females from the control group (17–18 days post emergence) and both unfed and blood fed mosquitoes from the second blood meal groups (17–18 days post emergence and 14 days after the 1^st^ blood meal). The result indicates that both protein spots were absent in old-aged female mosquitoes from at least day 17 post emergence. It suggests that the proteins may not be involved in blood feeding and transmission of malaria sporozoites in *An. camprestris*-like of the same age. However, it is interesting to identify and characterize these proteins in other mosquito species, whether they are involved in blood feeding and transmission of pathogens or not before any conclusions should be drawn.

To our knowledge, this study presents for the first time the salivary gland proteins that are involved in the second blood feeding on the day corresponding to the transmission period of the sporozoites to new mammalian hosts. The consequence of the changes of the proteins suggests that these proteins not only facilitated blood feeding but may play a role in malaria parasite transmission to the a new host. In particular the four proteins that were depleted more at second blood feed compared to the first are of interest: serine/threonine-protein kinase rio3; cyclophilin A, putative; short form D7r1; and gSG6 protein. The little that is known regarding the function of these proteins in blood feeding is discussed above. It should be noted that these changes are correlations and do not prove any direct function in enhancing transmission, the changes in composition are dynamic and complex, and there is no obvious reason why they should be useful for transmission. However, their role can be tested in functional analyses of malarial-infected salivary glands of *An. campestris*-like and other mosquito vectors and this would test their association with parasite transmission and the physiological processes that occur during blood feeding.

## Materials and Methods

### Mosquitoes and Blood Feeding

*An. campestris*-like colonies were successfully maintained for many consecutive generations in an insectary at the Department of Parasitology, Faculty of Medicine, Chiang Mai University, Thailand and were used in this study [44], [45]. The mosquitoes were reared and maintained in the insectary at 27±2°C with 70±10% relative humidity, and a photo-period of 12∶12 (light/dark) hours. Adult mosquitoes were given continuous access to a 10% sucrose solution. The mosquitoes were divided into two groups, the first blood meal group and the second blood meal group. For the first blood meal, three to four day old sugar-fed mosquitoes were allowed to feed on blood from immobilized mice and those that had fed to repletion were separated from the cohort and transferred to a new cage. Artificial mating was required for this particular species and was performed on the mosquitoes fed to repletion after the first blood meal except for those used as the first blood meal group which were dissected immediately after taking the meal. A second blood meal was given after 14 days (as the sporogonic cycle of *Plasmodium* in *Anopheles* mosquitoes takes about 14 days) and the females that fed a second time were collected and dissected immediately for salivary glands as described below. Salivary glands of unfed mosquitoes from each group were collected and used to compare with the blood-fed ones. During the entire procedure, a cup filled with water was placed in all the cages to facilitate oviposition. Tripicate experiments were performed on different cohorts and generations of mosquitoes.

### Ethical Clearance

The protocols were approved by the Animal Ethics Committee of the Faculty of Medicine, Chiang Mai University, Chiang Mai, Thailand.

### Salivary Gland Dissection

The female mosquitoes were cold anaesthetized on ice before salivary gland dissection. Salivary glands of the mosquitoes were dissected in phosphate-buffered saline [PBS; 10 mM Na_2_PO_4_, 145 mM NaCl (pH 7.2)] using fine entomological needles under a stereoscopic microscope at 4X magnification. The salivary glands were transferred to a microcentrifuge tube with a small volume of PBS and stored at −80°C until use.

### Two-dimensional Gel Electrophoresis

Two-dimensional gel electrophoresis was performed using the 2D system (GE Healthcare, UK). A Micro BCA Protein Assay Kit (Pierce, USA) was used for quantification of proteins. The total salivary gland protein content of female mosquitoes at three to five days after emergence was on the average 1.38±0.01 µg/gland pair [15] (70 gland pairs ≈97 µg). Therefore, in each experimental sample, 70 pairs of female salivary glands were used. The salivary glands were extracted and desalted using a 2-D Clean-Up kit (GE Healthcare, UK). Each pellet sample was solubilized in a 125 µl sample solubilization solution (8 M urea, 50 mM DTT, 4% CHAPS, 0.2% 3/10 Bio-lyte Ampholyte, 0.002% Bromophenol Blue) and then loaded on an IPG strip (pI 3–10, 7 cm, GE Healthcare, UK) to perform the first dimension isoelectric focusing (IEF) separation. Following 13 h of rehydration, the strips were focused using Ettan IPGphor III (GE Healthcare, UK) according to the manufacturer’s instructions. The focused IPG strips were then incubated in ten ml SDS equilibration buffer (6 M urea, 2% SDS, 0.05 M Tris, pH 8.8, 30% glycerol, 0.002% Bromophenol Blue) containing 100 mg dithiothreitol (DTT) for 15 min and for a further 15 min in 10 ml of equilibration buffer containing 250 mg iodoacetamide. The equilibrated strips were applied to the surface of vertical 15% SDS-polyacrylamide gels and proteins separated in the second dimension using the Mini-PROTEAN Tetra Electrophoresis System (Bio-Rad, USA). Protein molecular weight markers (Bio-Rad, USA) were applied to each gel.

### Coomassie Brilliant Blue (CBB) Staining and Gel Image Analysis

Following the electrophoresis, gels were Coomassie Brilliant Blue (CBB) stained. First, the gels were fixed in 50% methanol and 10% acetic acid for 30 min, then stained with 1% CBB in 10% methanol and 5% acetic acid for two h, and finally de-stained in 10% methanol and 5% acetic acid until dark protein bands were visible. The gels were scanned with the Imagescanner III (GE Healthcare, UK). A bioinformatics program (Image Master 2D Platinum, GE Healthcare, UK) was used to detect the number of spots in each gel and measure the molecular weight, the isoelectric point, and expression volume of each spot.

### Protein Quantification and Statistical Analysis

Each 2-DE sample was subjected to triplicate runs. Quantification of the average spot density (ASD) for each protein on 2-DE gels was carried out using the Image Master 2D Platinum software (GE Healthcare, UK). Statistical analysis (Student’s *t*-test, p≤0.05) was performed using SPSS 17.0 software (SPSS, Chicago, IL, USA) to compare of the average density of each protein spot during each feeding. Heat shock cognate (HSC) 70 was used as an internal control protein, based on previous work showing constitutive expression in *Ae. aegypti* salivary glands in response to heat shock [46] and no change in expression in response to blood feeding [9], and in *An. barbirostris* salivary glands no response to ageing [40].

### In-gel Digestion

Protein spots of interest were excised from the 2-D gels using sterile surgical blades with aseptic technique. The gel pieces were subjected to in-gel digestion using an in-house method developed by Proteomics Laboratory, National Center for Genetic Engineering and Biotechnology (BIOTEC), National Science and Technology Development Agency (NSTDA), Thailand [47]. The gel plugs were dehydrated with 100% acetonitrile (ACN), reduced with 10 mM DTT in 10 mM ammonium bicarbonate at room temperature for one hour and alkylated at room temperature for one hour in the dark in the presence of 100 mM iodoacetamide (IAA) in 10 mM ammonium bicarbonate. After alkylation, the gel pieces were dehydrated twice with 100% ACN for five minutes. To perform in-gel digestion of proteins, 10 µl of trypsin solution (10 ng/µl trypsin in 50% ACN/10 mM ammonium bicarbonate) was added to the gels followed by incubation at room temperature for 20 minutes, and then 20 µl of 30% ACN was added to keep the gels immersed throughout digestion. The gels were incubated at 37°C for a few hours or overnight. To extract peptide digestion products, 30 µl of 50% ACN in 0.1% formic acid (FA) was added into the gels, and then the gels were incubated at room temperature for 10 minutes in a shaker. Peptides extracted were collected and pooled together in a new tube. The pool extracted peptides were dried by vacuum centrifuge and kept at −80°C for further mass spectrometric analysis.

### NanoLC-MS Analysis

The protein digest was injected into an Ultimate 3000 LC System (Dionex, USA) coupled to an ESI-Ion Trap MS (HCT Ultra PTM Discovery System, Bruker, Germany) with electrospray at a flow rate of 300 nl/min to a nanocolumn (Acclaim PepMap 100 C18, 3 µm, 100A, 75 µm id ×150 mm). A solvent gradient (solvent A: 0.1% formic acid in water; solvent B: 80% 0.1% formic acid in 80% acetonitrile) was run for 40 min.

### Data Analysis and Protein Identification

Mass-Lynx was employed to generate peak lists (pkl files) from the raw data using the following parameters: (a) smooth windows (channels): 4.00, number of smooths: 2, smooth mode: Savitzky Golay; (b) percentage of peak height to calculate the centroid spectra, 80%; and (c) no baseline subtract was allowed. Mascot from Matrix Science Ltd. (London, U.K.) was used to search all of the tandem mass spectra [48]. The data was sent to the National Center for Biotechnology nonredundant (NCBInr) protein database. The search was performed taking other Metazoa as taxonomy. The other search parameters were enzyme of specificity strict trypsin; one missed cleavage; fixed modifications of Carbamidomethyl (C); oxidation (Met); peptide tolerance of 100 ppm; Fragment Mass Tolerance of ±0.5 Da; peptide change of 1+; and monoisotopic. Protein identification was made on the basis of Mowse score ≥30. All accession numbers of the best hit protein presented in this study is available online at http://www.ncbi.nlm.nih.gov.

## Supporting Information

Table S1 **A list of major protein spots decreased in volume immediately after the first blood feeding in female** ***An. campestris*** **-like salivary glands identified by NanoLC-MS, along with E-value and ion score.**
(XLS)
